# Morphological Parameters Associated with Ruptured Posterior Communicating Aneurysms

**DOI:** 10.1371/journal.pone.0094837

**Published:** 2014-04-14

**Authors:** Allen Ho, Ning Lin, Nareerat Charoenvimolphan, Mary Stanley, Kai U. Frerichs, Arthur L. Day, Rose Du

**Affiliations:** 1 Department of Neurosurgery, Brigham and Women's Hospital, Boston, Massachusetts, United States of America; 2 Harvard Medical School, Boston, Massachusetts, United States of America; 3 Department of Neurosurgery, University of Texas Medical School at Houston, Houston, Texas, United States of America; Children's National Medical Center, Washington, United States of America

## Abstract

The rupture risk of unruptured intracranial aneurysms is known to be dependent on the size of the aneurysm. However, the association of morphological characteristics with ruptured aneurysms has not been established in a systematic and location specific manner for the most common aneurysm locations. We evaluated posterior communicating artery (PCoA) aneurysms for morphological parameters associated with aneurysm rupture in that location. CT angiograms were evaluated to generate 3-D models of the aneurysms and surrounding vasculature. Univariate and multivariate analyses were performed to evaluate morphological parameters including aneurysm volume, aspect ratio, size ratio, distance to ICA bifurcation, aneurysm angle, vessel angles, flow angles, and vessel-to-vessel angles. From 2005–2012, 148 PCoA aneurysms were treated in a single institution. Preoperative CTAs from 63 patients (40 ruptured, 23 unruptured) were available and analyzed. Multivariate logistic regression revealed that smaller volume (p = 0.011), larger aneurysm neck diameter (0.048), and shorter ICA bifurcation to aneurysm distance (p = 0.005) were the most strongly associated with aneurysm rupture after adjusting for all other clinical and morphological variables. Multivariate subgroup analysis for patients with visualized PCoA demonstrated that larger neck diameter (p = 0.018) and shorter ICA bifurcation to aneurysm distance (p = 0.011) were significantly associated with rupture. Intracerebral hemorrhage was associated with smaller volume, larger maximum height, and smaller aneurysm angle, in addition to lateral projection, male sex, and lack of hypertension. We found that shorter ICA bifurcation to aneurysm distance is significantly associated with PCoA aneurysm rupture. This is a new physically intuitive parameter that can be measured easily and therefore be readily applied in clinical practice to aid in the evaluation of patients with PCoA aneurysms.

## Introduction

The guidelines for management of unruptured intracranial aneurysms remains one-dimensional even as more and more unruptured aneurysms undergo treatment [1]. As a result of the International Study of Unruptured Intracranial Aneurysms (ISUIA), treatment decision of unruptured intracranial aneurysms is currently based mainly on the size of the aneurysm [2]–[5]. However, a recent large prospective natural history study of unruptured aneurysms conducted by the Unruptured Cerebral Aneurysm Study (UCAS) of Japan has underscored the importance of not only size, but also the location and morphology of the aneurysm in predicting rupture risk [6]. Specifically, rupture risk was significantly elevated in aneurysms of the anterior and posterior communicating arteries, and even small aneurysms in these locations had a relatively high risk of rupture. Several groups including our own have begun to study contribution of morphological characteristics to the treatment decision of unruptured aneurysms in a systematic and location specific manner. Previous studies of large cohorts of mixed aneurysms have reported that variables such as the aspect ratio, undulation index, and size ratio are associated with ruptured aneurysms [7]–[9]. Looking at aneurysms in a location specific manner, our group found that aspect ratio, flow angle, and parent-daughter to be highly associated with middle cerebral artery aneurysm rupture [10]. Matsukawa et al. recently reported that rupture of anterior communicating artery aneurysms was associated with anterior dome projection, the presence of blebs, and size ≥5 mm [11].

Posterior communicating artery (PCoA) aneurysms are the second most common intracranial aneurysm and represent half of all internal carotid artery aneurysms [12]. Furthermore, though the rupture risk is similar to other anterior circulation aneurysms [13], smaller size alone in PCoA aneurysms does not necessarily correlate with decreased risk of rupture. In a review of PCoA aneurysms, the overall prevalence of aneurysms measuring less than 10 mm was 87.5%, and as many as 85.6% of ruptured PCoA aneurysms were less than 10 mm [14]. Thus, it is clear that size alone is not a reliable predictor of rupture risk and other physical characteristics of the aneurysm must be considered. We present a large sample of posterior communicating aneurysms that were assessed using a diverse array of morphological variables to determine the parameters associated with ruptured posterior communicating artery aneurysms.

## Methods

### Ethics Statement

The study was approved by the Brigham and Women's Hospital Institutional Review Board. Written consent from the patients was waived by the Institutional Review Board.

### Patient selection

The study population consisted of all patients with a diagnosis of posterior communicating artery (PCoA) aneurysm treated at the Brigham and Women's Hospital during a 7-year period between 2005 and 2012. Aneurysms that underwent reoperation, those that were associated with arteriovenous malformations, or those that lacked preoperative CT angiography (CTA) were excluded. Demographic and clinical information were collected from medical records. In particular, patient data on risk factors commonly associated with aneurysm development or aneurysm rupture were collected, including smoking status, family history, presence of multiple aneurysms, history of hypertension, and prior history of aneurysm rupture/SAH. The study was approved by the Institutional Review Board.

### Reconstruction of 3D models

**Figure 1:**
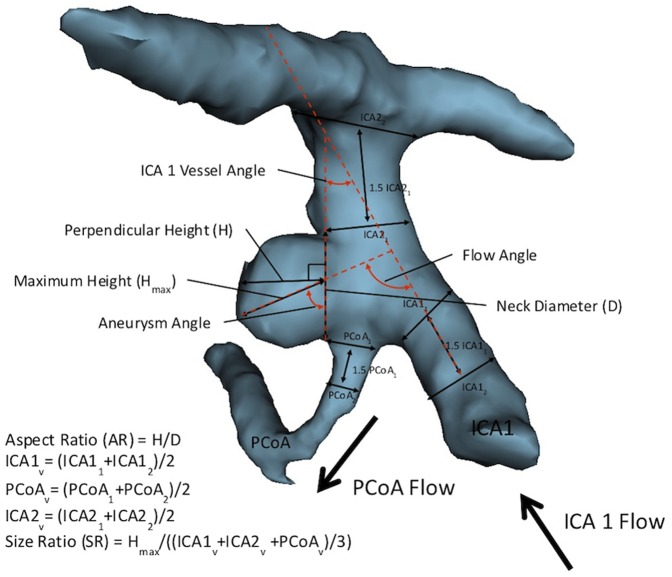
3D model of PCoA aneurysm depicting morphological variables previously studied in the literature. The aspect ratio (AR) is obtained by dividing the perpendicular height by the neck diameter. Size ratio (SR) is calculated by dividing the maximum height (H_max_) by the average composite diameter of the all vessels (ICA1_v_, PCoA_v_, ICA2_v_) involved with the aneurysm. Composite diameters are obtained by averaging the initial diameter of the vessel (ICA1_1_, PCoA_1_, ICA2_1_) at the aneurysm neck or branching point with the diameter of the vessel 1.5 away from the initial diameter (ICA1_2_, PCoA_2_, ICA2_2_). Aneurysm angle is defined as the angle between the vector formed by the maximum height of the aneurysm with the aneurysm neck. The vessel angle is defined as the angle between the vector of flow and the neck of the aneurysm. The flow angle is defined as the angle between the vector of flow and the vector formed by the maximum height of the aneurysm.

**Figure 2:**
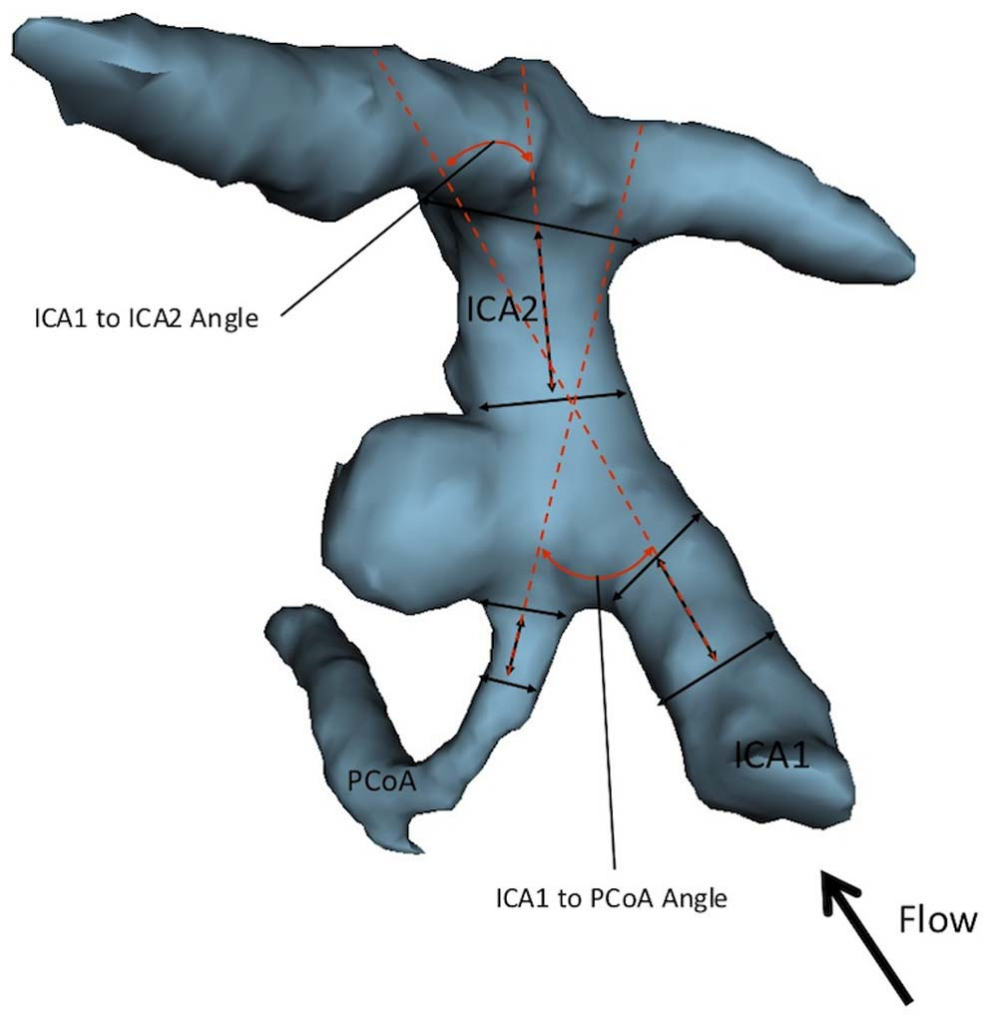
3D model of PCoA aneurysm depicting angular variables of the surrounding vasculature. There were three vessel-to-vessel angles measured. The ICA1 to ICA2 angle refers to the angle between the distal ICA (ICA2) and the proximal ICA (ICA1). The ICA1 to PCoA angle refers to the angle formed between the proximal ICA (ICA1) and PCoA.

As described in our prior study [10], we utilized 3D Slicer (referred as “Slicer” in the following text), an open source, multi-platform visualization and image analysis software [15], [16]. Pre-operative CT angiography (CTA) images were utilized to generate composite three-dimensional (3D) models of the aneurysm and surrounding vasculature. All CTAs were performed on a Siemens® SOMATOM Definition scanner with slice thickness of 0.75 mm and increment of 0.5 mm. We were able to separate the vascular compartment by thresholding. Aneurysm contours were then reconstructed using a triangle reduction and smoothing algorithm. This 3D surface model of the aneurysm and surrounding vessels could be manipulated freely in the Slicer environment. (Figures 1 and 2) Fiducial-based tractography was then utilized to manually measure volumes, lengths and angles in 3D space.

### Definition of morphological parameters

Morphological parameters examined in 3D aneurysm models included several variables already defined in the studies investigating other types of aneurysm (aneurysm size, aneurysm volume, aspect ratio, aneurysm angle, vessel angles, and size ratio, flow angles, and vessel-to-vessel angles)[7], [8], [10], [17] as well as several novel parameters that applied to the specific anatomy of the PCoA (distance from PCoA origin (or proximal neck of the aneurysm) to the internal carotid artery (ICA) bifurcation, angle between the proximal ICA (ICA1) and PCoA). (Figures 1 and 2) Briefly, aneurysm maximum height was utilized as an estimate of aneurysm size and refers to the largest cross-sectional diameter of the aneurysm measured from the base of the aneurysm from the reconstructed 3D model. The maximum perpendicular height was the height of the aneurysm as measured from the center of the base to the dome. Aspect ratio is the ratio of the maximum perpendicular height of the aneurysm to the average neck diameter of the aneurysm. Aneurysm angle is formed between the plane of the neck of the aneurysm and the vector of the maximum height of the aneurysm measured from the center of the aneurysm neck to furthest point on the dome of the aneurysm. It captures in the angle of inclination of the aneurysm from the plane of the neck.

The main surrounding vessels involved with the aneurysm include the ICA proximal to the aneurysm (ICA1), the ICA distal to the aneurysm (ICA2), and the PCoA. Size ratio is the ratio between the maximum aneurysm height and mean vessel diameters of all branches associated with the aneurysm. Specifically, the diameters of a particular vessel are determined by averaging the diameter of the cross-section at the neck of the aneurysm (D1) with the diameter of the cross-section at 1.5×D1 from the neck of the aneurysm. This average diameter was calculated for all vessels involved with the aneurysm to generate the composite mean vessel diameter utilized to calculate the size ratio. The vessel angle is angle between the respective vessel and the plane of the aneurysm neck. Centerlines for each vessel were determined by linking the two center points of the cross-sections utilized to measure the vessel diameter in size ratio. The ICA1/PCoA flow angle is the angle between the vector of maximum height of the aneurysm and the vector of centerline through the ICA1/PCoA that represents the vector of flow. This angle represents the angle at which the aneurysm is tilted with respect to the vector of flow through parent vessels. The new parameters devised specifically for PCoA aneurysms are described below. (Figures 1 and 2)

#### Angles

All previously described vascular angles including aneurysm, vessel, and vessel-to-vessel angles were measured with respect to the ICA1, ICA2, and PCoA. With respect to PCoA aneurysms, flow generally enters via the proximal ICA and divergences from the aneurysm into the PCoA and distal ICA. There were two vessel-to-vessel angles measured. (Figure 2) The ICA1 to ICA2 angle refers to the angle between the distal ICA (ICA2) and the proximal ICA (ICA1). This parameters measure the degree to which the blood flow through the proximal ICA (ICA1) must deviate in order to emerge into the distal ICA (ICA2). The ICA1 to PCoA angle refers to the angle formed between the proximal ICA (ICA1) and PCoA. This angle serves to measure the degree of blood flow deviation from the proximal ICA (ICA1) into the PCoA. The PCoA is not always well visualized, thus vascular angles for the PCoA are missing for many patients. Of note, the vessel-to-vessel angles are independent of the aneurysm itself and capture the context of the surrounding vasculature within which the aneurysm arises.

#### Distance to ICA Bifurcation

This parameter is specific to the anatomy of the PCoA and has not previously been investigated. The distance to the ICA bifurcation is defined by the distance from the proximal neck of the aneurysm and the bifurcation of the ICA into the middle cerebral and anterior cerebral arteries.

### Statistical Analysis

Differences in demographic and clinical characteristics by rupture status were examined using chi-square and two-tailed t-tests for binary and continuous variables, respectively. Univariate analysis was performed to compare the value of each morphological parameter between the ruptured and unruptured groups. Multivariate logistic regression was used to calculate the odds ratios (ORs) and 95% confidence intervals (95% CI) for the likelihood of aneurysm rupture after adjusting for age, sex, smoking status, family history, presence of multiple aneurysms, hypertension, and prior history of SAH. Morphological variables pertaining to the PCoA were excluded from this analysis since the PCoA was poorly visualized in a majority of patients. Subgroup analysis was subsequently conducted in patients with visualized PCoAs. Again, multivariate logistic regression was conducted with clinical and morphological variables, this time including the PCoA parameters. Statistical significance was defined as a type I error less than 0.05. Multivariate logistic regression was also conducted to create a model for predicting presence of intracerebral hemorrhage. This analysis included morphological variables pertaining to the aneurysm itself, and its relation to the internal carotid artery. All statistical analyses were performed using JMP Pro 10, SAS version 9.2 (SAS Institute Inc, Cary, North Carolina) and Excel 2007 (Microsoft Corp., Redmond, WA).

## Results

### Univariate Analysis

From 2005–2012, 148 PCoA aneurysms were treated in a single institution, and preoperative CTAs from 63 patients were analyzed. There were a total of 40 ruptured and 23 unruptured aneurysms. Demographic and clinical data is provided in Table 1. The mean age was 55.86±14.2 years. Patients with unruptured aneurysms were slightly older (means of 58.09 years unruptured vs 54.58 years ruptured), though this difference was not statistically significant. Fifty-four (86%) patients were women, and the gender compositions of the two arms of our study were similar (86% unruptured, 85% ruptured). Patients with unruptured aneurysms were significantly more likely to have multiple aneurysms or a history of prior aneurysm rupture (p = 0.034). Ruptured aneurysms were associated shorter distance to ICA bifurcation in a relationship that approached significance (11.3 mm unruptured versus 10.4 mm ruptured, p = 0.076). (Table 2) Rupture was also associated with larger ICA1 to ICA2 angle, and ICA1 to PCoA angle but these trends were not statistically significant. Larger ICA1 to ICA2 angle was significantly associated with lateral projection of the aneurysm (lateral projection 85.8 degrees vs not lateral projection 73.5 degrees, p = 0.013), linking the orientation of the surrounding vasculature with the direction and development of the aneurysm dome. Finally, PCoA aneurysms with lateral projection were more associated with rupture, and this relationship also approached significance (30% of unruptured vs 55% of ruptured, p = 0.052). Aneurysm sizes, as measured by maximal aneurysm height were similar between unruptured and ruptured aneurysms (5.85 mm unruptured vs 5.97 mm ruptured, p = 0.441).

**Table 1:** Baseline demographic and clinical data.

	Unruptured (n = 23)	Ruptured (n = 40)	*p* value
Age: Mean (SD)	58.09 (11.7)	54.58 (15.4)	0.156
Female sex (%)	20(86%)	34(85%)	0.831
Smoking	12 (52.2%)	26 (66.7%)	0.258
Hypertension	9 (39.1%)	16 (45.7%)	0.620
Family history	3 (13.6%)	2 (6.3%)	0.358
**Multiple aneurysms**	15 (65.2%)	15 (37.5%)	**0.034**
**History of prior rupture**	3 (13%)	0	**0.033**

**Table 2:** Univariate analysis for rupture.

	Unruptured (n = 23)	Ruptured (n = 40)	*p* value
Age	54.6 (15.4)	58.1 (11.7)	0.156
Female sex	87%	85%	0.830
Smoking	52.2%	66.7%	0.259
Hypertension	39.1%	45.7%	0.612
Family history	6.3%	13.6%	0.363
**Multiple aneurysms**	65.2%	37.5%	**0.033**
Volume (mm3)	115.2 (133.2)	98.8 (79.3)	0.313
Neck diameter (mm)	4.4 (1.5)	4.3 (1.3)	0.429
Aneurysm max height (mm)	5.9 (2.9)	6.0 (2.3)	0.441
Aneurysm angle	94.6 (22.9)	93.7 (26.4)	0.444
Aspect ratio	1.1 (0.6)	1.2 (0.5)	0.364
Lateral projection	30%	55%	0.052
Size ratio	1.8 (1)	1.9 (0.9)	0.360
Distance to ICA bifurcation (mm)	11.3 (2.3)	10.4 (1.9)	0.076
ICA1 vessel angle	52.8 (36.5)	50.1 (22.5)	0.385
PCoA vessel angle	54.2 (26.6)	50.7 (22.1)	0.373
ICA1 flow angle	106.4 (22.8)	102.4 (34.1)	0.306
PCoA flow angle	88.1 (31.5)	93.7 (47.9)	0.368
ICA1 to ICA2 angle	77 (19.2)	81.5 (20.9)	0.220
ICA1 to PCoA angle	98.8 (19.4)	113.3 (29.3)	0.080

### Multivariate Analysis

Multivariate logistic regression was conducted with the inclusion of known clinical risk factors for aneurysm rupture including age, gender, smoking, hypertension, family history, and presence of multiple aneurysms. A model was generated with these risk factors and the relevant morphological variables pertaining to the aneurysm and its relation to the ICA (Table 3). PCoA variables were not included in multivariate analysis because the PCoA was poorly visualized in the majority of patients. Our model revealed that a smaller volume, larger aneurysm neck diameter, and shorter ICA bifurcation to aneurysm distance were the most strongly associated with aneurysm rupture after adjusting for all other clinical and morphological variables (volume OR 0.98, 95% CI 0.95–0.99, p = 0.011; aneurysm neck diameter OR 3.52, 95% CI 1.01–17.9, p = 0.048; ICA bifurcation distance OR 0.44, 95% CI 0.19–0.80 p = 0.005).

**Table 3:** Multivariate analysis for rupture.

	Odds Ratio (95% CI)	*p* value
Age	0.95 (0.84–1.05)	0.347
Female sex	2.9 (0.2–64.36)	0.441
Smoking	0.33 (0.29–2.47)	0.287
Hypertension	3.51 (0.35–57.23)	0.295
Family history	1.85 (0.04–93.54)	0.743
Multiple aneurysms	0.20 (0.01–1.66)	0.145
**Volume (mm^3^)**	0.98 (0.95–0.99)	**0.011**
**Neck diameter (mm)**	3.52 (1.01–17.9)	**0.048**
Aneurysm max height (mm)	2.13 (0.37–15.73)	0.404
Aneurysm angle	1 (0.96–1.03)	0.812
Aspect ratio	0.92 (0.03–22.77)	0.960
Size ratio	0.65 (0.01–44.63)	0.838
**Distance to ICA bifurcation (mm)**	0.44 (0.19–0.80)	**0.005**
ICA1 vessel angle	1 (0.97–1.03)	0.865
ICA1 flow angle	0.99 (0.95–1.02)	0.501
ICA1 to ICA2 angle	1.02 (0.97–1.06)	0.490

Multivariate subgroup analysis was subsequently conducted in patients with visualized PCoA and included both clinical risk factors and morphological variables specific to the aneurysm and its relation to the PCoA (Table 4). Several parameters were significantly associated with rupture, including younger age (OR 0.71, 95% CI 0.03–0.98, p = 0.031), increased neck diameter (OR 79.5, 95% CI 1.74–1.5×10^12^, p = 0.018), and shorter ICA bifurcation to aneurysm distance (OR 0.04, 95% CI 5.78×10^−9^–0.59, p = 0.011).

**Table 4:** Multivariate analysis for rupture in the subgroup of aneurysms with visible PCoA.

	Odds Ratio (95% CI)	*p* value
**Age**	0.71 (0.03–0.98)	**0.031**
Smoking	7.04 (0.002–7.16×10^18^)	0.652
Hypertension	2.79 (4.9×10^−4^–4.07×10^3^)	0.730
Family history	7.34×10^10^ (0.74–.)	0.060
**Neck diameter (mm)**	79.5 (1.74–1.5×10^12^)	**0.018**
Aneurysm max height (mm)	0.49 (0.045–130)	0.565
**Distance to ICA bifurcation (mm)**	0.04 (5.78×10^9^–0.59)	**0.011**
ICA1 vessel angle	1.05 (0.88–1.36)	0.528
PCoA vessel angle	2.54 (6.96×10^−27^–2.65×10^12^)	0.920
ICA1 flow angle	1.7×10^−4^ (1.15×10^−16^–64.9)	0.206
PCoA flow angle	0.99 (0.87–1.95)	0.948

Of the 40 ruptured PCoA aneurysm included in our study, 9/40 presented with intracerebral hemorrhage on imaging. On univariate analysis, larger proximal ICA (ICA1) diameter (p = 0.023), shorter ICA bifurcation to aneurysm distance (p = 0.026), larger ICA1 to PCoA angle (p = 0.024), and lateral projection of ruptured PCoA aneurysms (p = 0.014) were significant independent predictors of intracerebral hemorrhage. (Table 5) Multivariate logistic regression demonstrated that the presence of intracerebral hemorrhage was significantly associated with male sex (female OR 7.5×10^−6^, 95% CI 6.8×10^−20^–0.5, p = 0.032), patients with lack of hypertension (hypertension OR 3.4×10^−6^, 95% CI 2.7×10^−4^–0.2, p = 0.009), aneurysms with lateral projection (OR 644, 95% CI 2.1–2.6×10^11^, p = 0.020), smaller volume (OR 0.9, 95% CI 0.6–0.98, p = 0.010), larger maximum height (OR 16.4, 95% CI 1.7–4.2×10^5^, p = 0.007), and smaller aneurysm angle (OR 0.9, 95% CI 0.6–0.99, p = 0.029). (Table 6)

**Table 5:** Univariate analysis for intracerebral hemorrhage.

	ICH (n = 9)	None (n = 31)	*p* value
Age	60.2	52.9	0.146
Female sex	77.8%	87.1%	0.507
Hypertension	37.5%	48.2%	0.594
Multiple aneurysms	55.6%	32.3%	0.210
**Lateral projection**	88.9% (8/9)	45.2% (14/31)	**0.014**
Volume (mm^3^)	77.2	106.0	0.135
Neck diameter (mm)	4.1	4.4	0.208
Aneurysm max height (mm)	6.1	5.9	0.388
Aneurysm angle	88.4	95.5	0.232
Aspect ratio	1.3	1.2	0.241
**ICA1 diameter (mm)**	3.6	2.9	**0.0231**
ICA1 vessel angle	41.6	53.0	0.106
ICA1 flow angle	107.6	100.6	0.223
Size ratio	2.0	1.9	0.385
**Distance to ICA bifurcation (mm)**	9.2	10.9	**0.026**
PCoA vessel angle	48.8	51.5	0.439
PCoA flow angle	81.8	98.0	0.309
ICA1 to ICA2 angle	82.9	81.0	0.382
**ICA1 to PCoA angle**	131.5	106.6	**0.024**

**Table 6:** Multivariate analysis for intracerebral hemorrhage.

	Odds Ratio (95% CI)	*p* value
Age	1.1 (0.9–2.2)	0.437
**Female sex**	7.5×10^−6^ (6.8×10^−20^–0.5)	**0.032**
**Hypertension**	3.4×10^−6^ (2.7×10^−4^–0.2)	**0.009**
Multiple aneurysms	1.9 (0.3–289)	0.748
**Lateral projection**	644 (2.1–2.6×10^11^)	**0.020**
**Volume (mm^3^)**	0.9 (0.6–0.98)	**0.010**
Neck diameter (mm)	18.3 (0.2–1.0×10^6^)	0.305
**Aneurysm max height (mm)**	16.4 (1.7–4.2×10^5^)	**0.007**
**Aneurysm angle**	0.9 (0.6–0.99)	**0.029**
Aspect ratio	97.0 (0.006–2.0×10^8^)	0.339
ICA1 diameter (mm)	0.06 (5.3×10^−6^–2.2)	0.129
ICA1 vessel angle	0.92 (0.76–1.02)	0.144
ICA1 flow angle	1.1 (0.96–1.7)	0.204

## Discussion

Posterior communicating artery aneurysms are among the most likely intracerebral aneurysms to rupture, and even smaller PCoA aneurysms that have not approached traditional size thresholds for intervention tend to rupture at a high rate [6], [14], [18]–[22]. Thus, further clinical and morphological parameters beyond size need to be delineated to assist in accurately predicting rupture risk when considering management options.

Because of the results from ISUIA, the size of the aneurysm has traditionally been the main determining factor for predicting rupture risk and treatment decisions for unruptured intracerebral aneurysms. However, follow-up studies have found that the majority of PCoA aneurysms rupture at smaller sizes than would be expected based on ISUIA [6], [14], [23]. This may represent the fact that flow induced hemodynamic stress involved at aneurysm site is important for rupture, independent of the size of the aneurysm itself [24]–[28]. In our study, aneurysm size as determined by maximal height was similar between ruptured and unruptured PCoA aneurysms. This is likely a result of treatment bias as larger unruptured PCoA aneurysms are more likely to undergo intervention. Given the matched aneurysm sizes between the two groups, the results of this study largely reflect the impact of morphological parameters unrelated to size.

In our prior study of morphologic characteristics of MCA aneurysms, we had divided morphologic features into 3 categories: morphology of the aneurysm itself, interaction between the aneurysm and associated vessels, and the relationship among the surrounding vasculature [10]. In our analysis of PCoA aneurysms, the most significant factors are the relationships of the surrounding vasculature.

Smaller ICA bifurcation to aneurysm distance was associated with ruptured aneurysms in both univariate and multivariate analyses. The mean difference between the two groups was almost one millimeter and represented a more than a 10% decrease in distance. The scenario of the ICA bifurcation being far away from the PCoA origin is akin to a middle cerebral artery (MCA) bifurcation aneurysm while the scenario of the ICA bifurcation being immediately after the aneurysm is somewhat geometrically similar to an anterior communicating artery (ACoA) aneurysm. This is consistent with the results of the UCAS study where anterior communicating artery aneurysms have a higher rupture rate than middle cerebral artery aneurysms [6]. Hemodynamic modeling of aneurysms may be helpful in explaining this phenomenon [29]–[32]. Fluid dynamics evaluation of the two geometries have demonstrated high wall shear stress at the branch point in the scenario of long ICA versus low wall shear stress between two branch points in the case of short ICA [29], [30]. In addition, low wall shear stress has been shown to be associated with aneurysm rupture [31]. Thus, while the difference in fluid dynamics between the two scenarios that contributes to rupture risk remains to be elucidated, the results are consistent with prior fluid dynamics results.

Ruptured aneurysms were also associated with larger ICA1 to ICA2 angles and ICA1 to PCoA angles but these trends were not statistically significant. A larger ICA1 to ICA2 angle means a sharper turn in the ICA. Aside from the ICA, the main daughter vessel influence on hemodynamics at the point of the aneurysm is conferred by the PCoA. The effect of a larger proximal ICA1 to PCoA angle is consistent with our prior study of MCA aneurysms where a sharper parent-daughter angle was significantly associated with ruptured aneurysms [10]. The greater the angle between the ICA1 and PCoA, the smaller the deviation of flow from the ICA1 parent vessel to the PCoA daughter vessel. This relationship can have a profound impact on the hemodynamics of flow and wall shear stress at the vascular branchpoint and aneurysm dome, and be linked to an increased risk of rupture [24], [30], [31], [33]–[35].

The anatomical relationship of the ICA and PCoA vessels and aneurysms within the supraclinoid segment of intracranial vasculature is both varied and complex, and their orientation has specific implications for the vascular and morphological parameters we considered. Yasargil described five categories of ICA-PCoA aneurysms according to the orientation of the aneurysm fundus: anterolateral, superolateral, superior postero-lateral (supratentorial), inferior posterolateral (infratentorial), and inferior posteromedial [36]. The most important configurations in modern practice relate to the lateral versus inferior projection of the aneurysm dome since inferiorly projecting aneurysms commonly compress the oculomotor nerve while laterally projecting aneurysms can be tightly adherent to the temporal lobe and are sources of temporal lobe hemorrhage when ruptured. Recent investigation has also shown that ruptured aneurysms are associated with lateral projection of the aneurysm dome [37]. This relationship held true within our sample, where ruptured aneurysms (55%) were more associated with lateral projection than unruptured aneurysms (30%). Aneurysms with lateral projection in our sample also had larger ICA1 to ICA2 angles. The larger the ICA1 to ICA2 angles in ruptured aneurysms may be associated with lateral projection of the aneurysm dome because of the sharper turns in the vasculature and larger deviations in parent to daughter flow. In this way, the surrounding vasculature must also be considered when analyzing anatomic relationships of the aneurysm.

### Intracerebral Hemorrhage

Intracerebral hemorrhage confers additional morbidity to aneurysm rupture, particularly for PCoA aneurysms where the hemorrhage is typically in the temporal lobe. Understanding the features that are associated with intraparenchymal hemorrhage would therefore be important in evaluating not only the risk of rupture but also the possible sequelae of a rupture. Lateral projection of the aneurysm dome is a well-known risk factor for intracerebral hemorrhage, and this was confirmed in our analysis [38]. The projections of these aneurysms into the temporal lobe make them much more likely to cause intracerebral hemorrhage upon rupture. Interestingly, smaller volume, larger maximum height, and smaller aneurysm angle were also significantly associated with intracerebral hemorrhage. Smaller volume and larger aneurysm maximum height implies a more elongated aneurysm which would be more likely to project into the temporal lobe and result in an intracerebral hemorrhage. The association of lack of hypertension with intracerebral hemorrhage may be because hypertension increases rupture risk at a smaller size [39], that is, prior to the aneurysm being large enough to project into the temporal lobe. Finally, the association with male sex does not have a clear morphological explanation and warrants further study.

### Limitations

There are a number of limitations to this study. This is a retrospective study of patients with PCoA aneurysms who were treated. There is therefore an inherent bias selection of the unruptured aneurysms. Also, all inferences made about the parameters examined can be associated with ruptured aneurysms only, and are not necessarily predictors of rupture risk. Furthermore, certain features of the aneurysm may be altered by the ruptured state. Nevertheless, the significant parameters in this study are largely that of the surrounding vasculature, which would not be altered by the rupture of an aneurysm. Finally, our model is a simplified one where material properties of the arterial wall, boundary conditions and other parameters that would affect the fluid dynamics were not taken into account. Measurements were performed manually taking advantage of the 3D slicer software fiducial tractography capabilities. Though significant steps have been made towards automated methods of assessing morphology to achieve greater consistency in measurement [40], we believe that measures made this way best recreate the applicability of our methodology in a clinical setting. This affords a clinician the ability to make measurements utilizing patient CTAs and open source software in an efficient manner.

## Conclusion

We performed a study dedicated to the morphological characteristics of posterior communicating artery aneurysms and found that ruptured aneurysms were significantly associated with shorter distance from the aneurysm to the internal carotid artery bifurcation. Furthermore, presentation with an intracerebral hemorrhage is associated with smaller aneurysm volume, larger aneurysm maximum height and smaller aneurysm angle, in addition to lateral projection, male sex, and lack of hypertension. Although they do not replace well-established clinical determinants of PCoA aneurysm rupture risk such as aneurysm size, these features do add an additional dimension of analysis that can be rapidly applied by clinicians when examining 3D reconstructions of unruptured aneurysms. These morphological associations are unique to the PCoA aneurysms and highlight the importance of location specific features when determining the natural history of aneurysms.

## References

[1] Huang MC, Baaj AA, Downes K, Youssef AS, Sauvageau E, et al (2011) Paradoxical trends in the management of unruptured cerebral aneurysms in the United States: analysis of nationwide database over a 10-year period. Stroke 42: 1730–1735. 21493902 10.1161/STROKEAHA.110.603803

[2] Komotar RJ, Mocco J, Solomon RA (2008) Guidelines for the surgical treatment of unruptured intracranial aneurysms: the first annual J. Lawrence pool memorial research symposium—controversies in the management of cerebral aneurysms. Neurosurgery 62: 183–193 discussion 193–184. 18300906 10.1227/01.NEU.0000311076.64109.2E

[3] Wiebers DO, Whisnant JP, Huston J 3rd, Meissner I, Brown RD Jr, et al (2003) Unruptured intracranial aneurysms: natural history, clinical outcome, and risks of surgical and endovascular treatment. Lancet 362: 103–110. 12867109 10.1016/s0140-6736(03)13860-3

[4] Mocco J, Komotar RJ, Lavine SD, Meyers PM, Connolly ES, et al (2004) The natural history of unruptured intracranial aneurysms. Neurosurg Focus 17: E3. 10.3171/foc.2004.17.5.3 15633980

[5] Ecker RD, Hopkins LN (2004) Natural history of unruptured intracranial aneurysms. Neurosurg Focus 17: E4. 10.3171/foc.2004.17.5.4 15633981

[6] Morita A, Kirino T, Hashi K, Aoki N, Fukuhara S, et al (2012) The natural course of unruptured cerebral aneurysms in a Japanese cohort. N Engl J Med 366: 2474–2482. 22738097 10.1056/NEJMoa1113260

[7] Raghavan ML, Ma B, Harbaugh RE (2005) Quantified aneurysm shape and rupture risk. J Neurosurg 102: 355–362. 15739566 10.3171/jns.2005.102.2.0355

[8] Dhar S, Tremmel M, Mocco J, Kim M, Yamamoto J, et al (2008) Morphology parameters for intracranial aneurysm rupture risk assessment. Neurosurgery 63: 185–196 discussion 196–187. 18797347 10.1227/01.NEU.0000316847.64140.81 PMC2570753

[9] Rahman M, Smietana J, Hauck E, Hoh B, Hopkins N, et al (2010) Size ratio correlates with intracranial aneurysm rupture status: a prospective study. Stroke 41: 916–920. 20378866 10.1161/STROKEAHA.109.574244

[10] Lin N, Ho A, Gross BA, Pieper S, Frerichs KU, et al (2012) Differences in simple morphological variables in ruptured and unruptured middle cerebral artery aneurysms. J Neurosurg 117: 913–919. 22957531 10.3171/2012.7.JNS111766

[11] Matsukawa H, Uemura A, Fujii M, Kamo M, Takahashi O, et al.. (2012) Morphological and clinical risk factors for the rupture of anterior communicating artery aneurysms. J Neurosurg. 10.3171/2012.11.JNS121210 23240701

[12] Ojemann RG, Crowell RM (1988) Internal carotid artery aneurysms. Surgical Management of Cerebrovascular Disease. 2nd ed. Baltimore: Williams and Wilkins. pp. 179–198.

[13] Clarke G, Mendelow AD, Mitchell P (2005) Predicting the risk of rupture of intracranial aneurysms based on anatomical location. Acta Neurochir (Wien) 147: 259–263 discussion 263. 15662565 10.1007/s00701-004-0473-3

[14] Forget TR Jr, Benitez R, Veznedaroglu E, Sharan A, Mitchell W, et al (2001) A review of size and location of ruptured intracranial aneurysms. Neurosurgery 49: 1322–1325 discussion 1325–1326. 11846931 10.1097/00006123-200112000-00006

[15] Pieper S, Halle M, Kikinis R (2004) 3D SLICER. Proceedings of the 1st IEEE International Symposium on Biomedical Imaging: From Nano to Macro 1: 632–635.

[16] Pieper S, Lorensen B, Schroeder W, Kikinis R (2006) The NA-MIC Kit: ITK, VTK, Pipelines, Grids and 3D Slicer as An Open Platform for the Medical Image Computing Community. Proceedings of the 3rd IEEE International Symposium on Biomedical Imaging: From Nano to Macro: 698–701.

[17] Ujiie H, Tamano Y, Sasaki K, Hori T (2001) Is the aspect ratio a reliable index for predicting the rupture of a saccular aneurysm? Neurosurgery 48: 495–502 discussion 502–493. 11270538 10.1097/00006123-200103000-00007

[18] Akimura T, Abiko S, Ito H (1991) True posterior communicating artery aneurysm. Acta Neurol Scand 84: 207–209. 1950462 10.1111/j.1600-0404.1991.tb04939.x

[19] Carter BS, Sheth S, Chang E, Sethl M, Ogilvy CS (2006) Epidemiology of the size distribution of intracranial bifurcation aneurysms: smaller size of distal aneurysms and increasing size of unruptured aneurysms with age. Neurosurgery 58: 217–223 discussion 217–223. 16462474 10.1227/01.NEU.0000194639.37803.F8

[20] Freytag E (1966) Fatal rupture of intracranial aneurysms. Survey of 250 medicolegal cases. Arch Pathol 81: 418–424. 5933816

[21] Hademenos GJ, Massoud TF, Turjman F, Sayre JW (1998) Anatomical and morphological factors correlating with rupture of intracranial aneurysms in patients referred for endovascular treatment. Neuroradiology 40: 755–760. 9860129 10.1007/s002340050679

[22] Prestigiacomo CJ, He W, Catrambone J, Chung S, Kasper L, et al (2009) Predicting aneurysm rupture probabilities through the application of a computed tomography angiography-derived binary logistic regression model. J Neurosurg 110: 1–6. 18928360 10.3171/2008.5.17558

[23] He W, Hauptman J, Pasupuleti L, Setton A, Farrow MG, et al (2010) True posterior communicating artery aneurysms: are they more prone to rupture? A biomorphometric analysis. J Neurosurg 112: 611–615. 19747044 10.3171/2009.8.JNS08731

[24] Burleson AC, Strother CM, Turitto VT (1995) Computer modeling of intracranial saccular and lateral aneurysms for the study of their hemodynamics. Neurosurgery 37: 774–782 discussion 782–774. 8559308 10.1227/00006123-199510000-00023

[25] Hashimoto N, Handa H, Nagata I, Hazama F (1980) Experimentally induced cerebral aneurysms in rats: Part V. Relation of hemodynamics in the circle of Willis to formation of aneurysms. Surg Neurol 13: 41–45. 7361257

[26] Kerber CW, Imbesi SG, Knox K (1999) Flow dynamics in a lethal anterior communicating artery aneurysm. AJNR Am J Neuroradiol 20: 2000–2003. 10588134 PMC7657773

[27] Nakatani H, Hashimoto N, Kang Y, Yamazoe N, Kikuchi H, et al (1991) Cerebral blood flow patterns at major vessel bifurcations and aneurysms in rats. J Neurosurg 74: 258–262. 1988596 10.3171/jns.1991.74.2.0258

[28] Tognetti F, Limoni P, Testa C (1983) Aneurysm growth and hemodynamic stress. Surg Neurol 20: 74–78. 6867933 10.1016/0090-3019(83)90112-x

[29] Ardakani SSJ, Jafarnejad M, Firoozabadi B, Saidi MS (2010) Investigation of wall shear stress related to factors in realistic carotid bifurcation geometries and different flow conditions. Transaction B: Mechanical Engineering 17: 358–366.

[30] Ujiie H, Liepsch DW, Goetz M, Yamaguchi R, Yonetani H, et al (1996) Hemodynamic study of the anterior communicating artery. Stroke 27: 2086–2093 discussion 2094. 8898821 10.1161/01.str.27.11.2086

[31] Fukazawa K, Ishida F, Umeda Y, Miura Y, Shimosaka S, et al.. (2013) Using computational fluid dynamics analysis to characterize local hemodynamic features of middle cerebral artery aneurysm rupture points. World Neurosurg. 10.1016/j.wneu.2013.02.012 23403347

[32] Celi S, Berti S (2013) Three-dimensional sensitivity assessment of thoracic aortic aneurysm wall stress: a probabilistic finite-element study. Eur J Cardiothorac Surg. 10.1093/ejcts/ezt400 23921161

[33] Ujiie H, Tachibana H, Hiramatsu O, Hazel AL, Matsumoto T, et al (1999) Effects of size and shape (aspect ratio) on the hemodynamics of saccular aneurysms: a possible index for surgical treatment of intracranial aneurysms. Neurosurgery 45: 119–129 discussion 129–130. 10414574 10.1097/00006123-199907000-00028

[34] Baharoglu MI, Schirmer CM, Hoit DA, Gao BL, Malek AM (2010) Aneurysm inflow-angle as a discriminant for rupture in sidewall cerebral aneurysms: morphometric and computational fluid dynamic analysis. Stroke 41: 1423–1430. 20508183 10.1161/STROKEAHA.109.570770

[35] Ford MD, Lee SW, Lownie SP, Holdsworth DW, Steinman DA (2008) On the effect of parent-aneurysm angle on flow patterns in basilar tip aneurysms: towards a surrogate geometric marker of intra-aneurismal hemodynamics. J Biomech 41: 241–248. 18078944 10.1016/j.jbiomech.2007.09.032

[36] Yasargil MG (1984) Microneurosurgery. New York: Thieme-Stratton.

[37] Matsukawa H, Fujii M, Akaike G, Uemura A, Takahashi O, et al (2014) Morphological and clinical risk factors for posterior communicating artery aneurysm rupture. J Neurosurg 120: 104–110. 24160476 10.3171/2013.9.JNS13921

[38] Yasargil MG (1984) Microneurosurgery: Thieme.

[39] Nahed BV, DiLuna ML, Morgan T, Ocal E, Hawkins AA, et al (2005) Hypertension, age, and location predict rupture of small intracranial aneurysms. Neurosurgery 57: 676–683 discussion 676–683. 16239879

[40] Piccinelli M, Steinman DA, Hoi Y, Tong F, Veneziani A, et al (2012) Automatic neck plane detection and 3D geometric characterization of aneurysmal sacs. Ann Biomed Eng 40: 2188–2211. 22532324 10.1007/s10439-012-0577-5

